# Geographically weighted regression of land cover determinants of *Plasmodium falciparum* transmission in the Ashanti Region of Ghana

**DOI:** 10.1186/1476-072X-13-35

**Published:** 2014-09-30

**Authors:** Lutz Ehlkes, Anne Caroline Krefis, Benno Kreuels, Ralf Krumkamp, Ohene Adjei, Matilda Ayim-Akonor, Robin Kobbe, Andreas Hahn, Christof Vinnemeier, Wibke Loag, Udo Schickhoff, Jürgen May

**Affiliations:** Bernhard-Nocht-Institute for Tropical Medicine, Research Group Infectious Disease Epidemiology, Hamburg, Germany; First Department for Internal Medicine, Division for Tropical Medicine, University Medical Centre Hamburg-Eppendorf, Hamburg, Germany; German Center for Infection Research (DZIF), Hamburg-Borstel-Lübeck, Kragujevac, Germany; Kumasi Centre for Collaborative Research in Tropical Medicine, Kumasi, Ghana; Komfo Anokye Teaching Hospital, Kumasi, Ghana; Animal Health and Food Safety Division, Animal Research Institute, Achimota-Accra, Ghana; Department of Paediatrics, University Medical Centre Hamburg-Eppendorf, Hamburg, Germany; Department of Geography, University of Hamburg, Hamburg, Germany

**Keywords:** Spatial epidemiology, Land use, Land cover, Malaria risk, Geographically weighted regression

## Abstract

**Background:**

Malaria is a mosquito-borne parasitic disease that causes severe mortality and morbidity, particularly in Sub-Saharan Africa. As the vectors predominantly bite between dusk and dawn, risk of infection is determined by the abundance of *P. falciparum* infected mosquitoes in the surroundings of the households. Remote sensing is commonly employed to detect associations between land use/land cover (LULC) and mosquito-borne diseases. Due to challenges in LULC identification and the fact that LULC merely functions as a proxy for mosquito abundance, assuming spatially homogenous relationships may lead to overgeneralized conclusions.

**Methods:**

Data on incidence of *P. falciparum* parasitaemia were recorded by active and passive follow-up over two years. Nine LULC types were identified through remote sensing and ground-truthing. Spatial associations of LULC and *P. falciparum* parasitaemia rate were described in a semi-parametric geographically weighted Poisson regression model.

**Results:**

Complete data were available for 878 individuals, with an annual *P. falciparum* rate of 3.2 infections per person-year at risk. The influences of built-up areas (median incidence rate ratio (IRR): 0.94, IQR: 0.46), forest (median IRR: 0.9, IQR: 0.51), swampy areas (median IRR: 1.15, IQR: 0.88), as well as banana (median IRR: 1.02, IQR: 0.25), cacao (median IRR: 1.33, IQR: 0.97) and orange plantations (median IRR: 1.11, IQR: 0.68) on *P. falciparum* rate show strong spatial variations within the study area. Incorporating spatial variability of LULC variables increased model performance compared to the spatially homogenous model.

**Conclusions:**

The observed spatial variability of LULC influence in parasitaemia would have been masked by traditional Poisson regression analysis assuming a spatially constant influence of all variables. We conclude that the spatially varying effects of LULC on *P. falciparum* parasitaemia may in fact be associated with co-factors not captured by remote sensing, and suggest that future studies assess small-scale spatial variation of vegetation to circumvent generalised assumptions on ecological associations that may in fact be artificial.

**Electronic supplementary material:**

The online version of this article (doi:10.1186/1476-072X-13-35) contains supplementary material, which is available to authorized users.

## Background

Malaria is the most common vector-borne infectious disease. In 2012, 207 million estimated malaria cases and an estimated 627,000 deaths occurred worldwide, 80% of them in Sub-Saharan Africa [1]. Distribution and incidence of vector-borne diseases are strongly influenced by spatial and temporal variation in the environment (e.g. weather [2], agriculture [3], urbanisation [4], hydrology [5]). It has been demonstrated in several studies that malaria risk varies over short distances [6–8]. These micro-scale spatial variations should be considered in public health interventions. In a previous study, we used a sub-group of infants from the current dataset to analyse spatial patterns of malaria [9]. We demonstrated that malaria incidence differs between and even within settlements, and that the risk of malaria decreases strongly with increasing distance of households to the forest fringe [9]; a finding that has been observed in other studies [10]. We also postulated that this might be due to higher exposure to mosquitoes on the outskirts of the village. The link of swamps and water bodies to malaria transmission has been demonstrated in various studies [11–14], since such habitats are ideal breeding grounds for mosquitoes. The two important vectors of *P. falciparum* in this region are *Anopheles gambiae* complex and *An. funestus* [15] *.* Both species have characteristic habitat preferences. The *An. gambiae* complex, comprising the main vectors, breed in small temporary habitats with little aquatic vegetation [16], but are known to be able to adapt to urban conditions [17], while *An. funestus* prefers more permanent water bodies for breeding [16].

In a recent hospital based study we used geographic information systems (GIS) and remote sensing data to describe environmental and temporal patterns of malaria incidence on village level in a rural area in Ghana. In this study, distinct cultivation in the proximity of homesteads was found to influence the risk of childhood malaria [12].

Although traditional regression analyses of spatial interactions often consider data captured on small-scale levels, they assume that such interactions are spatially homogenous throughout the study area [5, 18–20]. Such approaches cannot consider small-scale spatial variation and spatial autocorrelation. Albeit useful as a complimentary approach to describe spatially constant associations, they are insufficient to study spatially variable relationships.

Using data from the cohort presented in our previous publication [9] we aimed to explore possible associations between *P. falciparum* infection in infants and different land use/land cover (LULC) in the surroundings of their households. We investigated whether the previously described pattern of low malaria risk in village centres and higher risk in the surroundings was consistent throughout the study area, and explored whether it was based on an attenuating effect of built-areas, or an amplifying effect of specific land types bordering the settlements.

## Methods

### Study area

Malaria transmission is high (~130-140 cases/1000 per year in 2003–2005) in all of Ghana, with *P. falciparum* as the major species responsible for almost all infections [1]. This study was performed in nine villages of the rural Afigya-Sekyere District, Ashanti Region, Ghana. The total area of our study site covers approximately 200 km^2^. The vegetation of the study area is mainly semi-deciduous forest with major vegetation types of open forest, closed forest, wooded savannah, and various plantations. There is no spatial variation in altitude or climate. There are two wet seasons, from May to July, and during September. The dry season lasts from December to April. For the study region *P. falciparum* transmission is described to be perennial [15], with a transmission peak during the wet season [2].

### Study population and data collection

Data used for this study were collected in the frame of a randomized, placebo-controlled trial of intermittent preventive treatment of infants (IPTi) with sulfadoxine-pyrimethamine (SP) conducted between January 2003 and September 2005 (Trial registered at www.ClinicalTrials.gov: NCT00206739), and the findings of the original trial have been published [21]. A total of 1,070 infants were recruited at the age of 3 months and followed-up actively in monthly intervals, until the age of 24 months. In case of illness between active visits, the children’s parents were encouraged to visit the study team or one of the assigned health facilities in the district [21]. Blood samples were taken on every visit by finger-prick. These were screened for *P. falciparum* parasitaemia by microscopy according to standardized procedures described elsewhere [22]. At recruitment, data on predictors of *P. falciparum* infection (i.e. mosquito net/fly screen usage, ethnic group, age, financial situation of the household, education and occupation of the child’s mother, treatment with SP) were recorded using a standardized questionnaire. Detailed information on the study area and data collection has previously been published [9, 21, 23–25].

### Preparation of spatial data

We obtained one WorldView-2 image (acquired January, 4, 2011, in the dry season) to identify LULC characteristics of the study area. The data were available with 2 m spatial resolution and eight broad spectral bands (four standard colours, wavelengths: blue, 0.45-0.52 μm; green, 0.52-0.59 μm; red, 0.62-0.68 μm; and near-infrared 1, 0.77-0.86 μm, and four new colours, wavelengths: coastal, 0.40-0.45 μm, yellow, 0.58-6.25 μm, red edge, 7.05-7.45 μm, and near-infrared 2, 8.60-10.45 μm), along with one panchromatic band with 0.5 pixel/m. A Normalized Difference Vegetation Index (NDVI) image [NIR-red]/[NIR + red] was calculated as a measure for vegetation productivity. Different textural measures such as mean, contrast, entropy or correlation were computed from the panchromatic band. A new textural image for each textural measure was produced by moving several windows of different pixel areas (3x3 to 15x15) over the image. The optimal window size was determined by using a confusion matrix to assess the accuracy of the texture based classification. The textural images were then combined with the NDVI image and the eight spectral bands. All pre-processing steps to customize spatial data were carried out using ENVI 4.4 [26].

In March 2011, we conducted field sampling of LULC types using a Garmin eTrex^®^H Global Positioning System (GPS), ground-truthing 300 points randomly selected in the vicinity of the nine study villages, and by taking notes and photographs regarding the dominant vegetation. In a first step, we digitized reference areas and assigned them to one of the following nine LULC types:

-  banana/plantain plantation
-  built-up area: houses and buildings
-  cacao plantation
-  deforested area: burned, grassy or bushy underground, open spaces, and roads
-  forest: vegetation with dense tree cover and a closed canopy
-  orange plantation
-  palm tree plantation
-  swampy area: including floodplains, and crops belonging to the ground vegetation layer that are associated with flooded or wet soils (e.g. maize, eggplants, peppers, tomatoes)
-  water: river, stream or lake

Plantations of bananas/plantains, cacao, palm trees, and oranges were mostly mixed fields, but classified according to the most dominant crop. Polygons were created around each GPS point, defining the respective LULC. The combined bands were then classified using a supervised maximum likelihood classifier and a majority/minority analysis. For detailed information please see *Krefis et al.* [12].

For this study, we considered all eight broad spectral bands from the acquired multispectral WorldView-2 image along with the NDVI image. Additionally, the three textural images mean, contrast, and correlation with a window size of 9 × 9 pixels (equivalent to 9 × 9 m) had the highest accuracy of the texture-based classification and respective textural measures were chosen for the analyses accordingly (data not shown). By using reference areas for all nine LULC types we conducted a maximum likelihood classification of the combined NDVI image, the spectral, and the textural bands. Overall accuracy of the classification was 85%. We compared the results to other classification methods, such as Decision Tree-, Minimum Distance- and K-Means Classification [27, 28].

### Definition of outcome

To study the association of LULC characteristics and *P. falciparum* transmission we used the incidence rate of *P. falciparum* parasitaemia, defined as the number of episodes per person-year at risk as the outcome measure. Children were not considered to be at risk for 21 days after every episode of treated *Plasmodium* infection and/or after presumptive antimalarial therapy.

### Definition of exposure

We recorded the position of each household and added them to the LULC map using ArcGIS 10. Due to the young age of the study population and the fact that the vectors predominantly bite between dusk and dawn [29], we assumed that transmission mainly occurs within the household setting. The average flying range of the main vector *An. gambiae* is reported to be approximately 500 meters [18, 30]. Maximum flying range can be remarkably higher, but in general chances of mosquito movement sharply decreases with distance. Thus we applied a 500 meter buffer around each household location, and used the LULC within this buffer zone to define exposure. We categorized LULC according to its proportion of each household buffer. Since the proportions of each LULC type were unevenly distributed across the generated buffers, equally spaced categories (i.e. using quartiles) could not be applied. This would have resulted in either too narrow (too few observations) or too broad categories (too many observations), making analysis impossible. We therefore stratified the more abundant LULC types using 10% (if median proportion of all buffers > 1%, but < 15%), or 20% (if median proportion of all buffers > 15%) categories. Absence/presence stratification was used for those LULC types whose median proportion of all buffers was less than 1% (see Table 1 for more detail).

**Table 1:** **Proportions of land use/land cover types within a 500 m radius around each household**

Land use/land cover	Median	Minimum	Maximum	Interquartile range	Categories
Banana	19.0%	0.8%	36.2%	6.5%	0-20%, >20%
Built-up areas	33.7%	0%	81.7%	25.5%	0-20%, >20-40%, >40%
Cacao	0%	0%	47.1%	0%	Absence, presence
Deforested areas	10.1%	0.5%	42.2%	7.7%	0-10%, >10%
Forest	0%	0%	28.4%	0%	Absence, presence
Oranges	11.6%	0%	46.0%	13.3%	0-10%, >10-20%, >20%
Palm trees	5.2%	0%	63.6%	15.4%	0-10%, >10%
Swampy areas	9.6%	0.7%	36.6%	10.5%	0-10%, >10%
Water	0.6%	0%	3.4%	0.8%	Absence, presence

### Inclusion of variables

For the study population the following factors were shown to be associated with malaria in previous analyses of different sub-groups of the current data [5, 18, 24, 31]: IPTi with sulfadoxine-pyrimethamine, mosquito protection (none, bed nets/window screens), mother’s occupation (specifically farmer, non-farmer), mother’s basic education (primary school, at least secondary school), financial status of the family (poor, good), ethnic group (Akan, Northerners), and sickle cell trait (HbAS).

These variables were considered as potential confounders or co-factors of associations between LULC and *P. falciparum* infection. Thus, the association of each of the variables with the incidence of parasitaemia was analysed in univariate Poisson regression models using STATA 12 [32].

All variables that showed strong evidence for an association with parasitaemia (p-value < 0.05) in univariate models were considered as potential confounders or co-factors and included in all subsequent analyses. As the data were derived from a clinical trial it was decided *a priori* to include the treatment arm as well as the placebo arm in all models, irrespective of actual associations. LULC variables were stratified by their respective share of the buffer zone around the households, and were therefore associated by definition. Thus, we considered all LULC variables for further analyses.

### Geographically weighted regression

To assess the spatial variability of LULC influence on parasitaemia incidence we conducted a semi-parametric geographically weighted Poisson regression (GWPR), using the free software GWR 4.0 [33]. The dependent variable was parasitaemia episodes per person; the total time at risk was defined as the offset variable.

At first a global model was computed, assuming the process accounting for the disease to be spatially constant throughout the study area. Then a second model was constructed that assumed all LULC variables to vary locally. Based on the assumption of spatial autocorrelation, the local model constructed a spatial weights matrix, in which the individual observation was influenced by the surrounding observations. The extent of this influence was inversely related to distance. The ideal bandwidth of the matrix was determined by the small sample size bias corrected Akaike information criterion (AICc). The local model provided locally varying parameter estimates, standard errors, as well as the respective p-values (for more information on GWPR in disease mapping, refer to [34]). For the estimation of the local coefficients we used a fixed Gaussian kernel function.

Subsequently, a selection routine conducted a series of model comparison tests (i.e. AICc) for each local parameter in which, one by one, local (varying) terms were switched to global (constant) terms. If the subsequent model, assuming a spatially constant coefficient, outperformed the previous one assuming spatial variability, the variable was considered global for further analysis. This selection was repeated until there was no candidate left that could be changed, or no improvement was gained by changing local terms to global (for more information regarding the formulas, see formula in Additional file 1).

### Multi-collinearity

In regression analyses, and particularly in GWR, strong multi-collinearity can impair the model and produce artificial and erroneous effects ([35]). Multi-collinearity can never be entirely ruled out, but there are a few ways to test for this phenomenon. A measure of the degree of multi-collinearity is the variance influencing factor (VIF), defined as 1/(1-R^2^) of the regression model. There is, however, no consensus regarding the interpretation of VIF. A rule of thumb is that VIF of larger than 4–10 indicate strong multi-collinearity (for more information regarding multi-collinearity, refer to [36]). We conducted linear regression analyses for each the local coefficients as the dependent variable, and all other local coefficients as the independent variables. We chose a VIF of > 4 as our cut-off for multi-collinearity.

### Mapping of results

The relative risk (observed parasitaemia rate/expected parasitaemia rate) as well as the local regression coefficients and the respective p-values were mapped using the Geostatistical Analyst extension of ArcGis 10 [37]. We used the kernel smoothing method, using a Gaussian smoothing function (standard smoothing factor: 0.2), and the ideal bandwidth as identified by the GWPR (0.005049 degrees).

The average annual *P. falciparum* rate was categorized in 4 steps (0–1, >1-2, >2-3, >3). To compare the incidence rate ratios (IRR) of LULC classes, we used seven categories (strongly negative ≤0.33, moderately negative >0.33-0.66, weakly negative >0.66-0.83, none >0.83-1.2, weakly positive >1.2-1.5, moderately positive >1.5-3, strongly positive >3). The significance level was categorized in three classes (>0.05, 0.05-0.01, <0.01) of which only the least two categories were displayed on the map.

### Ethics statement

Aims and principles of the study were explained in detail to participants and informed consent was obtained by signature or thumb print by the caregiver. The Committee on Human Research, Publications, and Ethics, School of Medical Sciences, Kwame Nkrumah University of Science and Technology, Kumasi, Ghana approved the study design and the informed consent procedures.

## Results

### Study population

In total, 1,070 infants were recruited for this study. All individuals were followed up monthly and tested for *P. falciparum* parasitaemia by microscopy. Geographic coordinates of the households were available for 1,050 individuals. Only individuals with available data for all variables identified as associated with malaria were included in the analysis (n = 878). Study characteristics by village are presented in (Additional file 1: Table S5).

### Parasitaemia

**Figure 1:**
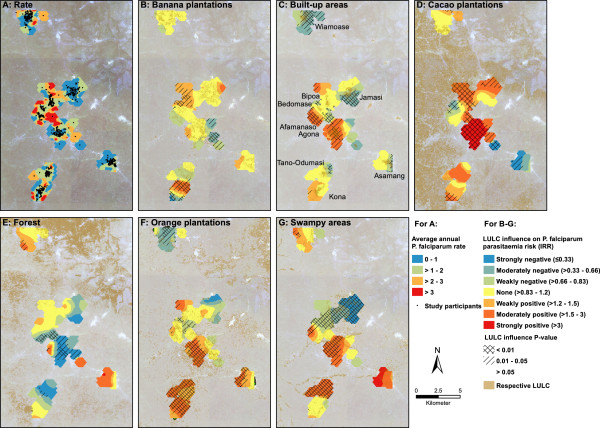
**Average annual** ***P. falciparum*** **rate of the study participants (A).** Local incidence rate ratios (colour), p-values (hatching), and area covered by the respective land use/land cover of spatially varying parameters (**B** = banana plantations, **C** = built-up areas with town names, **D** = cacao plantations, **E** = forest, **F** = orange plantations, **G** = swampy areas). Visualisation of all parameters was conducted via kernel smoothing, using a Gaussian smoothing function (factor = 0.2), and a bandwidth of 0.005049 degrees.

In total, 17,008 observations were included in the analysis. The total time at risk was 1,485.84 person-years, with a median of 511 days at risk per individual (IQR = 113 days). A total of 3,857 parasitaemia episodes were recorded. The parasitaemia rate was 3.2 per person-year at risk (95% CI: 3.1-3.3). In 64 individuals no episode of parasitaemia was diagnosed during follow-up. Assuming the study population is representative for the overall population, the rates of *P. falciparum* parasitaemia appeared to be lowest in population centres and highest on the outskirts (and less populated towns) (see Figure 1A).

### Classification and distribution of LULC variables

Built-up areas were the most abundant LULC within the vicinity of the households, with a median proportion of 33.7% of the defined buffers (see Table 1). We therefore stratified built-up areas into three categories (0-20%, > 20-40%, >40%). Orange plantations had a median proportion of 11.6% of the buffers, and were stratified into three categories (0-10%, >10-20%, >20%). The median proportion of swampy areas within the buffers was 9.6%, resulting in two categories (0-10%, >10%). Cacao plantations, forest, and water all had a median buffer-proportion of below 1% and were thus categorised as absence/presence.

Deforested areas (median proportion: 10.1%) and palm trees (median proportion: 5.2%) were grouped into two categories of 0-10% and >10%, whereas banana plantations (median proportion: 19.0%) were grouped into two categories of 0-20% and >20% (for proportion of LULC of household buffers by village, see Additional file 1: Table S6; for sample size per category, see Additional file 1: Table S7).

### Association of potential confounders/co-factors with parasitaemia

The univariate Poisson regression showed that - apart from sickle cell trait (HbAS) - all variables that were associated with clinical malaria in previous analyses [21, 24, 25] were also associated with the incidence of parasitaemia in general (Table 2). Therefore, ethnic group, education and occupation of the mother, mosquito protection, and financial situation were included in subsequent analyses, together with the treatment group, as defined *a priori*.

**Table 2:** **Risk factors of** ***P. falciparum*** **parasitaemia assessed by univariate Poisson regression**

Variable	Incidence rate ratio	P-value (Walds test)	95% confidence interval	
Ethnic group: Northerners	1.16	0.001	1.06	1.27
Financial situation^¥^	0.72	<0.001	0.67	0.78
Mosquito protection^¤^	0.78	<0.001	0.72	0.84
Mother education^£^	0.72	<0.001	0.67	0.77
Mother occupation^¢^	1.55	<0.001	1.45	1.67
SP-arm^μ^	0.91	0.003	0.85	0.97
Sickle cell trait (HbAS)	0.97	0.56	0.88	1.07

Categories: ^¥^good financial situation, ¤uses bed-net/fly-screen, ^£^at least secondary school education, ^¢^mother works as a farmer, ^μ^IPTi with sulfadoxine-pyrimethamine.

### Semi-parametric geographically weighted Poisson regression

In a first step, a global model was generated, i.e. the influence of all independent variables was considered spatially homogenous. This model (see Table 3) yielded an AICc of 3193. Built-up areas were negatively associated with *P. falciparum*, with an IRR of 0.80 (95% CI: 0.74-0.86) per 20% increase in built-areas within the surroundings of the household. Orange plantations were positively associated with parasitaemia, with an IRR of 1.10 (95% CI: 1.03-1.17) per 10% area covered by this LULC type. There was no evidence that other LULC variables were associated with parasitaemia in this global model. Of the previously described influencing factors, mosquito protection (IRR: 0.84, 95% CI: 0.78-0.91), a higher financial status (IRR: 0.83, 95% CI: 0.76-0.90), and a basic school education of the child’s mother (IRR: 0.83, 95% CI: 0.77-0.89) were associated with a lower *P. falciparum* parasitaemia rate, whereas the child’s mother working as a farmer was associated with a higher rate (compared to other occupations: IRR: 1.38, 95% CI: 1.28-1.48). IPTi with SP showed a protective effect on *P. falciparum* parasitaemia (IRR: 0.93, 95% CI: 0.87-0.99).

**Table 3:** **Global model of a semi-parametric geographically weighted Poisson regression, assuming all parameters are spatially homogenous**

Variable	Incidence rate ratio	95% confidence interval		P-value
Intercept	0.01	0.01	0.02	<0.001
Banana^¶^	1.06	0.99	1.15	0.10
Built-up areas^¶^	0.80	0.74	0.86	<0.001
Cacao^†^	0.98	0.88	1.09	0.68
Deforested areas^§^	1.01	0.93	1.10	0.83
Forest^†^	0.94	0.84	1.05	0.26
Oranges^§^	1.10	1.03	1.17	<0.01
Palm trees^§^	0.99	0.90	1.10	0.91
Swampy areas^§^	0.98	0.90	1.07	0.60
Water^†^	0.96	0.87	1.06	0.45
Ethnic group: Northerners	1.02	0.92	1.13	0.68
Financial situation^¥^	0.83	0.76	0.90	<0.001
Mosquito protection^¤^	0.84	0.78	0.91	<0.001
Mother’s education^£^	0.83	0.77	0.89	<0.001
Mother’s occupation^¢^	1.38	1.28	1.48	<0.001
SP-arm^μ^	0.93	0.87	0.99	0.019

Categories: ^§^10%, ^¶^20%, ^†^not present/present, ^¥^good financial situation, ^¤^uses bed-net/fly-screen, ^£^at least secondary school education, ^¢^mother works as a farmer, ^μ^IPTi with sulfadoxine-pyrimethamine.

In the next step, the semi-parametric GWPR was conducted. This model was an improvement to the global model, as the AICs decreased from 3193 to 2460, and was therefore considered for further interpretation. The ideal bandwidth was calculated to be 0.005049 degrees (approx. 550 m). Taking spatial variability into account revealed that the influence of plantations of oranges, cacao and bananas, as well as forest, swampy and built-up areas varied substantially throughout the study area (see Table 4). Particularly the impact of an increase in both swampy areas and orange plantations on *P. falciparum* parasitaemia varied considerably (see Figure 1F + G). The influence of swampy areas ranged from strongly negative in the more urban regions to moderately positive in the more riverine areas (median IRR/category: 1.15). Orange plantations had a moderately negative influence in the north, but a moderately positive influence in the south of the study area (median IRR/category: 1.11). The impact on *P. falciparum* parasitaemia associated with an increase in land covered by banana plantations (median IRR/category: 1.02) ranged from strongly negative (IRR <0.33) to moderately positive (IRR: 1.5-3), however values for most areas were not statistically significant. The influence of an increase in built-up areas was also mixed, with (moderately) positive and (moderately) negative associations in areas within close proximity (median IRR/category: 0.94). Cacao plantations showed extreme variations, but an increase in land covered by this LULC type was predominantly positively associated with parasitaemia risk (median IRR/category: 1.33), whereas the effect of forest was mainly protective (median IRR/category: 0.90).

**Table 4:** **Final model of the semi-parametric geographically weighted Poisson regression, with global parameters (top) and local parameters (bottom)**

Global variable	Incidence rate ratio	95% confidence interval		P-value
Deforested areas^§^	0.88	0.78	0.99	0.032
Palm trees^§^	0.86	0.71	1.03	0.11
Water^†^	1.16	0.94	1.44	0.17
Ethnic group: Northerners	1.08	0.95	1.22	0.26
Financial status^¥^	0.82	0.76	0.90	<0.001
Mosquito protection^¤^	0.82	0.76	0.90	<0.001
Mother’s education^£^	0.89	0.82	0.96	0.003
Mother’s occupation^¢^	1.34	1.24	1.45	<0.001
SP-arm^μ^	0.92	0.86	0.98	0.013
**Local variable**	**Median incidence rate ratio**	**Minimum incidence rate ratio**	**Maximum incidence rate ratio**	**Interquartile range**
Intercept	0.01	0.00	0.10	0.02
Banana^¶^	1.02	0.29	1.76	0.25
Built-up areas^¶^	0.94	0.51	2.11	0.46
Forest^†^	0.90	0.05	2.95	0.51
Cacao^†^	1.33	0.17	6.21	0.97
Oranges^§^	1.11	0.27	2.21	0.68
Swampy areas^§^	1.15	0.22	3.59	0.88

Categories: ^§^10%, ^¶^20%, ^†^not present/present, ^¥^good financial situation, ^¤^uses bed-net/fly-screen, ^£^at least secondary school education, ^¢^mother works as a farmer, ^μ^IPTi with sulfadoxine-pyrimethamine.

The influence of deforested areas, palm trees, and water did not vary significantly across the study area. These variables remained in the model as global terms. When accounting for the spatially variable influence of the other LULC types, it appeared that deforested areas had a spatially homogenous association with lower *P. falciparum* parasitaemia incidence rates (IRR: 0.88, 95% CI: 0.78/0.99). For all other variables the association or lack of association with the *P. falciparum* parasitaemia remained unchanged.

### Multi-collinearity

The VIF for each local LULC, as derived from the respective linear regression model, showed no signs of strong multi-collinearity. The values ranged from 1.5 for swampy areas to 3.9 for forest. The mean VIF of all models was 2.6.

## Discussion

Most environmental studies assume a consistent influence of covariates [5, 18, 19, 38], even though this may not always be appropriate [34]. In the current analysis, we demonstrate that the model fit of a regression model on associations between LULC and incidence of *P. falciparum* parasitaemia improves substantially by assuming spatial variability of the LULC variables. The analysis revealed that the effect of built-up areas and orange plantations on *P. falciparum* parasitaemia observed in the global model varied strongly across the study area with IRR ranging from 0.51-2.11 for built-up areas, and 0.27-2.21 for orange plantations. In addition, the strongly varying effects of swampy areas, cacao plantations, and forest were masked by the assumptions made by the global model that detected no associations at all. Furthermore, the final model, accounting for the spatial variability of other LULC variables, revealed a spatially homogenous attenuating effect of deforested areas on *P. falciparum* parasitaemia (IRR: 0.88, 95% CI: 0.78-0.99).

**Figure 2:**
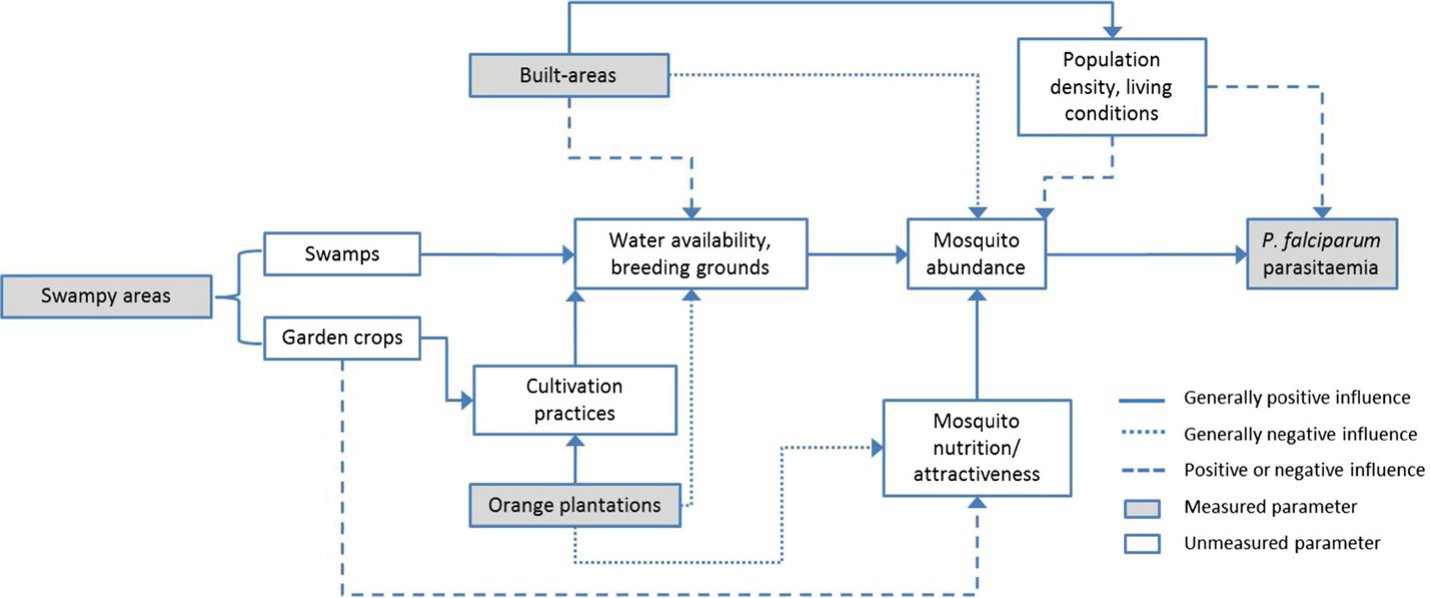
**Potential causal diagram of selected LULC variables to** ***P. falciparum parasitaemia.***

When assessing associations between environmental variables and *P. falciparum* parasitaemia one must consider the causal pathway on which the variables under study lie. It needs to be distinguished between variables with a direct influence on *P. falciparum* parasitaemia (e.g. mosquito abundance), those that are associated with conditions that have a direct influence (e.g. mosquito breeding grounds), and proxies or confounders (e.g. orange plantations that may be associated with irrigation). Allowing spatial heterogeneity within the regression model allows clearer interpretation regarding the true nature of potential associations. This study showed that analyses assuming spatial homogeneity are insufficient to study environmental relationships, because they fail to differentiate between a non-existing association of LULC and *P. falciparum* incidence, and strongly positive and negative associations within the same area that may be mediated through different causal pathways. Potential causal pathways of built-up areas, orange plantations, and swampy areas to *P. falciparum* parasitaemia are summarized in Figure 2.

While several studies suggest a positive association between deforested areas and mosquito abundance or malaria risk [39, 40], these studies do not distinguish between areas that were cleared for farmland (categorized separately in our study), and areas being transformed into sealed urban areas, such as roads and parking lots [41]. While farmland is often irrigated and may thus increase malaria risk, urbanized areas offer less potential breeding grounds, and homesteads closer to the fringe of the village may act as a barrier for the vectors, possibly explaining the negative association observed in this study.

This hypothesis is supported by other studies that described an attenuating effect of built-up/urban areas on the incidence of malaria [4, 42, 43]. While the global model suggests such an attenuating influence of built-up areas on parasitaemia risk, the association is too heterogeneous to be generalised (see Figure 1C), and requires further investigation.In contrast, the spatial heterogeneity of swampy areas is easily explained. In our study, swampy area is a broad LULC type that included swamps, floodplains, and crops belonging to the ground vegetation layer. It is therefore not surprising that the influence of swampy areas on parasitaemia incidence is inconsistent throughout the study area. A strong association between swampy areas and parasitaemia risk exists in the riverine south of the study region. Here, it is mainly floodplains and the nearby swamps (see Figure 1G) that offer ideal breeding grounds for mosquito vectors. In the central part, floodplains and swamps are less prominent, but here it is the more fragmented ground vegetation layer (garden crops, e.g. eggplants, peppers, tomatoes) that predominantly constitutes the LULC type. Such soils hold less stagnant moisture and are hence less suitable for mosquito breeding. Unfortunately, it was not possible to distinguish these plants from actual swamps by means of remote sensing.

The GWPR showed that the influence of orange plantations on parasitaemia risk varied substantially throughout the study area, ranging from moderately positive in the southern parts to moderately negative in the north. On first thought, positive values were surprising as the breeding habitat demands of both vectors strongly differ from the sandy and therefore dry soils found in orange plantations. Orange peels and their oil are traditionally used a natural insect repellent [44, 45], and a study investigating the attraction of *An. gambiae* mosquitoes to various fruits and seedpods concluded that oranges do not act as natural attractors [46]. However, as with swampy areas, the strong association of orange plantations and parasitaemia risk was predominantly found in the riverine south of the study area, indicating a possible interaction between the two LULC types. Furthermore, the density of orange plantations in the southern parts suggests more intense cultivation, possibly associated with agricultural trenches, irrigation, or the use of organic fertilizers (see Figure 1F), indicating that this LULC may in fact be a proxy for predictors of elevated parasitaemia risk that were not captured by remote sensing. The positive influence of irrigation and other agricultural practices on malaria prevalence has been described in various studies [3, 47–49]. Although we observed positive associations between banana plantations and childhood malaria in another region in Ghana [12], this finding was not confirmed in this study. The effect of banana plantations on parasitaemia shows strong variations thus making interpretation difficult (Figure 1B). It appears as though the influence of banana plantations followed a similar pattern as the effect of orange plantations and may also be associated with irrigation or farming practices.The influence of cacao plantations and forests varied extensively throughout the study area (Figure 1D + E). However, these findings should be interpreted with caution, considering the low abundance of both LULC classes in the vicinity of the households (median < 1%).

Surprisingly, water bodies were not clearly associated with malaria risk in the present study. However, the main vector of *P. falciparum* in the study area (*An. gambiae*) is known to prefer small, temporary breeding habitats such as hoof prints, puddles, or inundated land that would not be identified via remote sensing. As the last precipitation event occurred more than 6 weeks before the satellite images were taken, the true abundance of water bodies surrounding the households was likely underestimated, possibly masking an association with the risk for parasitaemia.

We used the same data presented here for a previous analysis [9] identifying spatial patterns of malaria incidence on the household level. However, our current analysis design differs distinctly. First, the outcome of this study was defined as *P. falciparum* parasitaemia instead of clinical malaria. Parasitaemia is a more stable indicator for the exposure to infected mosquito bites, as the risk of developing clinical malaria is influenced by genetic factors (e.g. sickle-cell trait), as well as acquired immunity [50–52]. Second, theoretically, intermittent preventive treatment with sulfadoxine-pyrimethamine protects against malaria but not necessarily parasitaemia. As parasitaemia is a more adequate indicator of *P. falciparum* infection than clinical malaria, we did not restrict the analysis to the placebo group of the study, as done in the previous analysis. We instead adjusted for treatment arm in the GWPR model.

In a further previous study [12] we employed the current method to investigate LULC determinants for the risk of childhood malaria on the village level in a rural area in Ghana. We applied various buffer ranges (0.5 km, 1 km, 1.5 km, and 2 km), and received similar results for all ranges. While this analysis assumed spatial stationarity of all variables, we used GWPR in the current study to assess whether and how the effects vary in space. Since the aim of the current study was to investigate variations on a small-scale, we decided to only use a 500 m buffer range around the households. A problem encountered in both studies is that the satellite images did not allow for a differentiation between swamps, and plants belonging to the ground vegetation layer (e.g. eggplants, tomatoes, peppers), resulting in an ecologically diverse LULC type of possibly contrary influences on malaria risk.

The major limitation of our study is the time span of approximately six years between health records and LULC data. Hence, the surrounding LULC might have changed since the health data were collected. Maximum likelihood procedures were used for supervised classification of land cover data, with a classification accuracy of 85%. This method gave more accurate results than other classification methods such as Decision Tree-, Minimum Distance- or K-Means Classification (data not shown). A comparison of satellite images from Google Earth (from 2003, [53]) and those we used (2011) showed a slight trend of urbanisation, with an increase in houses and deforested areas, and a slight decrease in forest areas (data not shown). Therefore, in our analyses, the proportion of built-up land is likely to have been overestimated, consequently underestimating its attenuating effect on *P. falciparum* parasitaemia.

To better interpret spatial associations of LULC and mosquito-borne diseases, future studies using similar approaches must be more theory driven with regard to classifying LULC classes. Since this study primarily grouped LULC types based on similar characteristics in the remote sensing images, certain LULC types had a high ecological diversity (e.g. swampy areas). We encourage that future studies consider habitat ecology of crops and soil types when classifying LULC types, with particular focus on the water storing capabilities.

## Conclusion

This study demonstrates that the association between LULC and parasitaemia risk is not spatially homogenous; a false assumption that weakens most study designs investigating this topic. Particularly the influence of built-up areas, swampy areas, and orange plantations on malaria risk was highly variable. These observations would have been masked by traditional regression analysis. While the exact reasons for variation require further ecological investigation, it is important to comprehend that ecosystems and their interactions are too complex to be modelled under the assumption of spatial stationarity. We therefore encourage considering spatial variability in further studies, particularly focusing on small-scale landscape or habitat heterogeneity.

## Electronic supplementary material

Additional file 1: Table S5: Population size, annual *P. falciparum* parasitaemia rate, and known influencing factors by village. **Table S6:** Land use/land cover proportion within a 500 m buffer around the study participants’ households, by village. **Table S7:** Sample size for each land use/land cover category in the study region. (DOCX 24 KB)

## Abbreviations

AICc: Akaike information criterion
CI: Confidence interval
GIS: Geographic information systems
GPS: Global positioning system
GWPR: Geographically weighted poisson regression
IRR: Incidence rate ratio
IQR: Interquartile range
IPTi: Intermittent preventive treatment of infants
LULC: Land use/land cover
NDVI: Normalized difference vegetation index
SP: Sulfadoxine-pyrimethamine
VIF: Variance influencing factor.
