# Supplementary material for: Geographically weighted regression of land cover determinants of Plasmodium falciparum transmission in the Ashanti Region of Ghana
Source: Int J Health Geogr. 2014 Sep 30;13:35. doi: 10.1186/1476-072X-13-35 (PMC4192530; doi:10.1186/1476-072X-13-35)
Supplement: Supplementary file 1 — Additional file 1: Table S5: Population size, annual P. falciparum parasitaemia rate, and known influencing factors by village. Table S6: Land use/land cover proportion within a 500 m buffer around the study participants’ households, by village. Table S7: Sample size for each land use/land cover category in the study region. (DOCX 24 KB) [file 12942_2014_603_MOESM1_ESM.docx]

**Supplementary material:**

Table 5: Population size, annual *P. falciparum* parasitaemia rate, and known influencing factors by village.

|  | | | Afamanaso | Agona | Asamang | Bedomase | Bipoa | Jamasi | Kona | Tano-Odumasi | Wiamoase | |
| --- | --- | --- | --- | --- | --- | --- | --- | --- | --- | --- | --- | --- |
| Population size | | | 2508 | 9321 | 5277 | 1492 | 3875 | 9096 | 5853 | 3453 | 12,877 | |
| Annual *P. falciparum* rate | | | 5.1 | 2.7 | 2.6 | 4.2 | 4.9 | 2.1 | 2.8 | 3.9 | 2.7 | |
| Participants (n) | | | 50 | 118 | 102 | 36 | 104 | 107 | 121 | 101 | | 139 |
| Male/female | | | 30/20 | 62/56 | 51/51 | 21/15 | 57/47 | 58/51 | 60/61 | 49/53 | | 62/77 |
| Ethnic group* | Akan | | 47 | 92 | 97 | 36 | 102 | 75 | 109 | 87 | | 125 |
|  | Northerners | | 3 | 26 | 5 | 0 | 2 | 32 | 12 | 14 | | 14 |
| Financial status ¥ | | | 10 | 30 | 14 | 13 | 18 | 32 | 18 | 36 | | 71 |
| Beta-globin gentotype* | | HbAA | 32 | 88 | 77 | 26 | 76 | 80 | 85 | 84 | | 103 |
|  |  | HbAC | 9 | 14 | 7 | 3 | 4 | 12 | 15 | 10 | | 16 |
|  |  | HbAS | 5 | 11 | 14 | 5 | 13 | 12 | 17 | 5 | | 13 |
| Mosquito protection ¤ | | | 35 | 107 | 79 | 25 | 73 | 92 | 87 | 95 | | 126 |
| Mother’s education £ | | | 35 | 81 | 85 | 26 | 75 | 75 | 88 | 72 | | 95 |
| Mother’s occupation ¢ | | | 25 | 18 | 24 | 5 | 32 | 9 | 12 | 25 | | 41 |
| SP arm µ | | | 25 | 57 | 47 | 16 | 53 | 62 | 64 | 50 | | 69 |
| Categories: ¥ good financial situation, ¤ uses bed-net/fly-screen, £ at least secondary school education, ¢ mother works as a farmer, µ treatment with sulfadoxine-pyrimethamine, * smaller groups not mentioned | | | | | | | | | | | | |

Table 6: Land use/land cover proportion within a 500m buffer around the study participants' households, by village.

|  | Afamanaso | Agona | Asamang | Bedomase | Bipoa | Jamasi | Kona | Tano-Odumasi | Wiamoase |
| --- | --- | --- | --- | --- | --- | --- | --- | --- | --- |
|  | Median (IQR) | Median (IQR) | Median (IQR) | Median (IQR) | Median (IQR) | Median (IQR) | Median (IQR) | Median (IQR) | Median (IQR) |
| Banana ¶ | 18.1 (4.7) | 18.5 (9.0) | 17.8 (3.3) | 17.1 (1.8) | 18.5 (4.3) | 22.8 (2.9) | 15.8 (3.6) | 23.6 (4.9) | 19.4 (5.5) |
| Built-up areas ¶ | 17.9 (5.8) | 56.6 (21.8) | 39.2 (14.5) | 14.5 (1.3) | 23.4 (9.4) | 42.3 (21.0) | 39.6 (15.6) | 31.5 (8.6) | 44.5 (21.1) |
| Cacao † | 0.0 (0.1) | 0.0 (0.0) | 0.0 (0.0) | 0.0 (0.3) | 0.0 (0.1) | 0.0 (0.0) | 0.0 (0.3) | 0.0 (0.0) | 0.0 (0.1) |
| Deforested areas § | 17.3 (6.8) | 5.6 (3.9) | 5.7 (1.3) | 12.4 (2.3) | 9.7 (3.1) | 5.2 (1.6) | 10.6 (2.6) | 12.8 (1.8) | 25.3 (9.1) |
| Forest † | 0.0 (0.1) | 0.0 (0.0) | 0.0 (0.0) | 0.0 (0.0) | 0.0 (0.0) | 0.0 (0.0) | 0.0 (0.1) | 0.0 (0.0) | 0.5 (1.4) |
| Oranges § | 21.9 (4.1) | 8.5 (9.3) | 6.8 (4.1) | 29.9 (2.9) | 13.1 (3.8) | 5.0 (3.7) | 16.0 (7.0) | 19.9 (9.7) | 5.2 (5.4) |
| Palm trees § | 13.8 (14.0) | 1.0 (2.0) | 11.3 (13.0) | 19.8 (5.0) | 21.6 (9.6) | 4.3 (7.3) | 8.7 (6.9) | 2.5 (2.6) | 0.1 (0.7) |
| Swampy areas § | 6.5 (2.3) | 5.4 (3.2) | 17.7 (2.9) | 6.1 (1.1) | 11.5 (6.2) | 17.9 (9.6) | 9.3 (4.6) | 6.1 (4.5) | 2.7 (5.6) |
| Water † | 0.1 (0.0) | 1.2 (0.4) | 0.8 (0.3) | 0.1 (0.1) | 0.4 (0.5) | 0.7 (0.2) | 0.5 (0.4) | 0.8 (0.3) | 0.0 (0.0) |
| Categories: §10%, ¶ 20%, † absence/presence | | | | | | | | | |

Table 7: Sample size for each land use/land cover category in the study region.

| Land use/land cover | Sample size per category (n=878) | | |
| --- | --- | --- | --- |
|  | 1 | 2 | 3 |
| Banana (20%) | 518 | 360 | n/a |
| Built-up areas (20%) | 202 | 334 | 342 |
| Cacao (y/n) | 724 | 154 | n/a |
| Deforested areas (10%) | 431 | 447 | n/a |
| Forest (y/n) | 717 | 161 | n/a |
| Oranges (10%) | 393 | 291 | 194 |
| Palm trees (10%) | 544 | 334 | n/a |
| Swampy areas (10%) | 476 | 402 | n/a |
| Water (y/n) | 224 | 654 | n/a |

**Formula:**

The following equation for a semi-parametric GWPR was used in our analysis:

$$\sigma_{i} \sim Poisson \left[ N_{i}\exp\left( \mu_{i} \right) \right]$$

$$\mu_{i}= \beta_{o}\left( u_{i}, v_{i} \right)+ \sum_{k=1}^{9} \beta_{k}\left( u_{i}, v_{i} \right)\alpha_{k,i}+ \sum_{l=1}^{6} z_{l}\delta_{l,i}$$

where $\sigma_{i}$ is the sum of parasitaemia episodes of the study participant at location $i$, $N_{i}$ is the time at risk at location $i$, $\alpha$ is the $k$th local variable with a local coefficient $\beta$, and $\delta$ is the $l$th global variable with the fixed coefficient $z$.

The formula used for the fixed Kernel bandwidth selection:

$$w_{ij}=\exp\left( -d_{ij}^{2}/\theta^{2} \right)$$

where $w_{ij}$ is the weight value of observation at location $j$ for estimating the coefficient at location $i$, $d_{ij}$ is the Euclidean distance between $i$ and $j$, and $\theta$ is a fixed bandwidth defined by a distance metric measure.
